# Genome-wide identification, phylogenetic, and expression analysis under abiotic stress conditions of Whirly (WHY) gene family in *Medicago sativa *L.

**DOI:** 10.1038/s41598-022-22658-3

**Published:** 2022-11-04

**Authors:** Qian Ruan, Yizhen Wang, Haoyu Xu, Baoqiang Wang, Xiaolin Zhu, Bochuang Wei, Xiaohong Wei

**Affiliations:** 1 College of Life Science and Technology, Gansu Agricultural University, Lanzhou, 730070 China; 2 Gansu Key Laboratory of Crop Genetic Improvement and Germplasm Innovation, Lanzhou, 730070 China; 3 Gansu Key Laboratory of Arid Habitat Crop Science, Lanzhou, 730070 China

**Keywords:** Biological techniques, Computational biology and bioinformatics

## Abstract

The WHY family is a group of plant-specific transcription factors, that can bind to single-stranded DNA molecules and play a variety of functions in plant nuclei and organelles, participating in the regulation of plant leaf senescence. It has been identified and analyzed in many species, however, the systematic identification and analysis of the *WHY* genes family have not yet been reported in alfalfa (*Medicago sativa *L.). Therefore, to explore the function of alfalfa the *WHY* genes, and 10 *MsWHY* genes were identified and further characterized their evolutionary relationship and expression patterns by analyzing the recently published genome of alfalfa. Comprehensive analysis of the chromosome location, physicochemical properties of the protein, evolutionary relationship, conserved motifs, and responses to abiotic stresses of the *WHY* gene family in alfalfa using bioinformatics methods. The results showed that 10 *MsWHY* genes were distributed on 10 chromosomes, and collinearity analysis showed that many *MsWHYs* might be derived from segmental duplications, and these genes are under purifying selection. Based on phylogenetic analyses, the *WHY* gene family of alfalfa can be divided into four subfamilies: I-IV subfamily, and approximately all the *WHY* genes within the same subfamily share similar gene structures. The 10 *MsWHY* gene family members contained 10 motifs, of which motif 2 and motif 4 are the conserved motifs shared by these genes. Furthermore, the analysis of cis-regulatory elements indicated that regulatory elements related to transcription, cell cycle, development, hormone, and stress response are abundant in the promoter sequence of the *MsWHY* genes. Real-time quantitative PCR demonstrated that *MsWHYs* gene expression is induced by drought, salt, and methyl jasmonate. The present study serves as a basic foundation for future functional studies on the alfalfa WHY family.

## Introduction

The small family of WHY proteins are single-strand DNA/RNA binding proteins located in organelles and nuclei with a characteristic "whirligig" secondary structure and a conserved KGKAAL DNA binding domain in angiosperms^1^. WHIRLY domains are comprised of four structural topologies that are characterized by two antiparallel four strands of β sheets enabled by a C-terminal helix-loop-helix motif^2,3^. Higher plants share a KGKAAL motif in the WHIRLY domain that mediates binding to single-strand DNA (ssDNA)^1^. In addition, WHY-like proteins with a high structural similarity but lacking the KGKAAL motif are also found in green algae^4^. Although algae have only one similar WHY protein, most plants in nature have two types of WHY proteins, WHY1 and WHY2^5^. WHY1 is a nuclear-localized protein that also targets chloroplasts^6^. WHY2 targets mitochondria and studies have shown that WHY2 is a protein that is triple localized between mitochondria, plastids, and the nucleus^7,8^. Although most plant species have two WHY proteins, there are three WHY proteins in Arabidopsis and other members of the cruciferous family, and WHY3 doubly targets are chloroplasts and mitochondria^8,9^. Desveaux et al. isolated the first WHY family member PBF2 (PR-10a Binding Factor 2) from potatoes in 2000. It binds to ERE (Activator Response Element) in a single strand, regulates the expression of the potato pathogen-related gene PR-10A, and transmits resistance signals, later named *StWHY1*^10^. Subsequently, members of the *WHY* gene family were found in Arabidopsis^8^, corn^11,12^, tomato^13^, and many others, suggesting that they may play a very important role in plant growth and physiology.

In recent years, as more and more studies have been conducted on the function of WHY proteins, it has been confirmed that these proteins have many important functions in plant development and stress tolerance. WHY1 acts as a transcription factor in the nucleus and is involved in regulating pathogen response pathways and the expression of downstream target genes for plant senescence, such as PR10a potatoes^10^, WRKY53 in Arabidopsis mustard^14^, and *HvS40* in driver proteins and barley^15^. In addition, WHY1 enrichment in cysticercoid membranes affects plant photosynthesis and redox stress^7,16,17^. For example, a reduced abundance of WHY1 can lead to delayed chloroplast development and leaf senescence^18,19^. WHY1 also has a significant effect on the synthesis and response of plant hormones related to plant growth and defense, such as ABA and SA. This is evidenced by studies of seed germination and senescence^20,21^. Meanwhile, WHY1, as a co-factor of homologous recombination and DNA double-strand break repair in organelles, maintains organelle genome stability and influences telomere maintenance and microRNA synthesis^22–24^. WHY1 also interacts with WHY3 to maintain organelle genomic stability and protein metabolism^17,22^. WHY2 acts as a DNA/RNA-binding protein in mitochondria and activates NAD1/CCB382 gene expression, and WHY2 binds to the promoter of SWEET11/15, which encodes sucrose transporter, in the nucleus^25^. It also increased the expression of genes involved in jasmonic acid signaling and related defense responses. These results suggest that WHY2 plays an important role in carbon redistribution between organelles and nuclei^26^. In addition, overexpression of WHY2 led to the accumulation of starch particles in the chloroplast of pericarp cells, leading to a phenotype of wilting, yellowing, and premature aging of leaves and horny fruits^26^. Although little has been reported about plant phenotypes overexpressing WHY1 or WHY3^27^, transgenic tomato lines overexpressing *SlWHY1* showed increased resistance to cryogenic stress by altering photosynthetic gene expression and modifying starch accumulation^28^. Arabidopsis plants that overexpress WHY2 exhibit early decay^22^. *MicroRNA840* (*miR840*) is a PPR and WHY3 protein that occurs only in Arabidopsis and can specifically target Arabidopsis through post-transcriptional gene silencing of PPR and WHY3^29^.

As a perennial legume forage of the genus Medicago, alfalfa has the characteristics of high yield, good palatability, and strong adaptability has a long cultivation history, and is widely planted^30^. Alfalfa can not only be used as fodder but also has the function of water and soil conservation, soil improvement, and ecological environment protection^31^. Therefore, cultivating tolerant alfalfa varieties is an economic and effective way to resist adversity environment. This experiment uses bioinformatics analysis to identify the alfalfa *WHY* gene family at the genome-wide level and further analyzes the gene structure, chromosome distribution, and promoter cis-acting elements. QRT-PCR was used to analyze *WHY* gene expression in alfalfa leaves at different treatment time points under salt, drought, and methyl jasmonate stress. This study can provide a theoretical basis for further research on *WHY* gene function in alfalfa.

## Materials and methods

### Identification and data collection of alfalfa WHY family

First, we obtained and downloaded alfalfa genome data from the Alfalfa Breeders Toolbox (https://www.alfalfatoolbox.org/)^32^. To identify all members of the alfalfa WHY family, we downloaded amino acid sequences from the TAIR database (http://www.arabidopsis.org/)^33^of the proposed southern WHY family (*AtWHY1*, *AtWHY2*, and *AtWHY3*) as decoys to retrieve the alfalfa genome database at the genome-wide level. WHY members have typical Whirly domains and can further be used in Pfam tools (http://pfam.xfam.org/family)^34^ to remove homologous sequences from canonical Whirly domains. Reuse online tools NCBI-CDD (https://www.ncbi.nlm.nih.gov/cdd/)^35^and SMART (http://smart.embl-heidelberg.de/)^36^to predict and identify all possible WHY family members in alfalfa. After deduplication, the genes left were considered alfalfa *WHY* genes.

### Basic physicochemical properties, secondary structure analysis, and 3D structure prediction of alfalfa *WHY* genes

To determine the physical and chemical parameters of each alfalfa WHY protein, the online software ExPASY (https://web.expasy.org/protparam/) was used to calculate the molecular weight (MW), isoelectric point (PI), amino acid numbers, and the average value of hydrophilicity (GRAVY)^37^. BUSCA was used to predict protein subcellular localization (http://busca.biocomp.unibo.it/)^38^. The secondary structure of proteins was predicted using the online tool SOPMA (http://npsa-pbil.ibcp.fr/cgi-bin/npsa_automat.pl?page=npsa_sopma.html.)^39^. Using online software ExPaSy SWISS-MODEL (https://swissmodel.expasy.org/interactive)for MsWHY proteins homology modeling for three-dimensional structure^40^.

### Protein interaction network diagram construction, phylogenetic and promoter cis-acting elements analysis of the *MsWHYs*

Based on the model plant *Arabidopsis thaliana*, the interaction of the alfalfa WHY protein network was predicted. The protein network structure diagram was constructed using STRING (http://STRINGdb.org/) software (confidence limit is 0.4)^41^. We used nine species to study the evolutionary relationship between alfalfa *WHY* genes and other plants’ *WHY* genes, including Arabidopsis (*Arabidopsis thaliana* L.), tobacco (*Nicotiana tabacum* L.), maize (*Zea mays* L.), barrel medic (*Medicago truncatula* L.), soybean (*Glycine max* L.), tomato (*Solanum lycopersicum* L.), grape (*Vitis vinifera* L.), sorghum (*Sorghum bicolor* L.), rice (*Oryza sativa* L.). Multiple alignments of the above nine species and alfalfa WHY protein sequences were performed by ClustalW of MEGA X^42^. The phylogenetic tree was constructed using the maximum-likelihood (ML) method of MEGA X, with 1000 bootstrap replications^42^. After the phylogenetic tree was constructed, the family members were classified according to the classification criteria of Arabidopsis, tobacco, and rice. The 2000 bp sequence upstream of the *WHY* gene was used as the promoter of the alfalfa *WHY* gene. Using PlantCARE (http://bioinformatics.psb.ugent.be/webtools/plantcare/html/)^43^database to predict the promoter cis-acting elements, and display them in the form of graphs.

### Gene structure and conserved motif analysis

WHY family DNA and CDS sequences were selected from alfalfa whole-genome sequencing and gene annotation files, respectively. Using the web-based bioinformatic tool GSDS2.0 (http://gsds.cbi.pku.edu.cn/index.php) to graphically display the exon/intron genomic structures of alfalfa *WHY* genes^44^. The conserved motifs of alfalfa WHY proteins were analyzed using Multiple Expectation Maximization for Motif Elicitation (MEME Suite) (http://meme-suite.org/)^45^, and the maximum number of patterns determined in the MEME program was adjusted to 10 and the width of the domain was set from 6 to 100^46^.

### Analysis of chromosome localization, gene duplication, and synteny of the *MsWHYs*

Chromosome mapping analysis of alfalfa *WHY* gene family using the MapInspect software (http://mapinspect.software.informer.com/)^47^. On the plant genome duplication database server (http://chibba.agtec.uga.edu/duplication/index/locket), the duplicate gene pairs are detected^48^. The non-synonymous substitution rate (Ka), synonymous substitution rate (Ks), and the Ka/Ks ratio were calculated using TBtools^49^. The *MsWHY* gene family synteny was analyzed using the One Step MCScanX tool in TBtools^49^.

### Planting and stress treatment of alfalfa material

*Medicago sativa* L. ‘Sandeli’ (Suntory scientific name M.sativa L., variety registration number 247, registration date 2002-12-11, applicant Chen Gu, etc., reporting unit Bailu (Tianjin) International Co., Ltd., variety type introduction) was used in this study. Alfalfa plants were grown in pots with a diameter of 15 cm, containing a mixture of nutrient soil and vermiculite (8:2 v/v), in a growth chamber at 25 ± 1 °C under a 12 h light and dark photoperiod. After alfalfa plants reached the age of 35 days, they were then randomly separated into three groups: PEG (drought stress), NaCl (salt stress), and MeJA (hormone treatment). Spray normal water as a control. The drought group and salt-treated group were irrigated with 50 mL 10% PEG and 100 mmol/L NaCl solution once after every 2 days for 7 days, respectively. With methyl jasmonic acid (200 mmol/L), respectively, the face of positive and negative of spraying alfalfa leaf, spray sterile water as control. The treatment time was 0 h, 3 h, 6 h, 9 h,12 h, 24 h, and 48 h. The mature leaves were sampled quickly and put into liquid nitrogen, 3 copies for each treatment, and then placed in − 80 °C cryopreservation for subsequent quantitative experiments by following the protocol mentioned.

### RNA extraction and qRT-PCR detection

Using Shenggong’s UNIQ-10 column Trizol total RNA extraction kit to extract total RNA from each sample, and use Nano-Drop 2000 UV spectrophotometer to detect RNA quality and concentration. Use M-Mu LV first-strand cDNA synthesis kit reverse transcription RNA to obtain cDNA. After detecting the concentration, uniformly dilute to 100 ng/ul as the qRT-PCR reaction template. Using NCBI Primers for qPCR were designed by primer-BLAST tools (www.ncbi.nlm.nih.gov/tools)^50^. The primers used in the experiment are shown in the S1 Table. Use Shenggong’s 2 × SG. Fast qPCR Master Mix kit, the reaction system is 20 μl, the PCR reaction program is 95 °C pre-denaturation for 10 min, and then 40 cycles including 95 °C denaturation for 15 s and 60 °C annealing for 1 min, the instrument used for Applied Biosystems 7500, the experimental results are processed by the 2^−ΔΔCt^ method^51^. Each experiment was repeated three times with independent RNA samples. Analysis of variance (ANOVA) of the relative expression level of each gene at different sampling points under each abiotic stress treatment was carried out following a generalized linear model using SPSS statistical software. Significant differences in mean values at different sampling times were determined by Tukey’s pairwise comparison tests, as indicated by different letters in the figures. The graphical representation of the experimental findings was produced by using Graphpad.

### Ethical approval and consent to participate

This study does not include human or animal subjects. All experimental studies and experimental materials involved in this research are in full compliance with relevant institutional, national and international guidelines and legislation.

## Results

### Basic physicochemical properties and secondary structure analysis

Accurate identification and an unified nomenclature are essential for future research into the *WHY* gene family in alfalfa. Here, we identified a total of 10 *WHY* genes from the alfalfa genome and named them from *MsWHY1* to *MsWHY8* based on their chromosomal location, with *MsWHY5.1*-*MsWHY5.3* being homologous (Table 1). The protein lengths varied greatly from 91 aa (*MsWHY1*) to 290 aa (*MsWHY2*). The molecular weights ranged from 10,490.38 to 32,491.09 D, and the PI variation ranged from 5.82 (*MsWHY2*) to 10.28 (*MsWHY1*). The calculated grand average of hydropathy index (GRAVY) values of all *MsWHYs* was − 0.076 to − 0.388, indicating that they were hydrophilic in nature. The determination of the subcellular localization of MsWHY proteins will help to understand the molecular function. The subcellular localization prediction showed that *MsWHY1* and *MsWHY2* were located in mitochondria, *MsWHY4*, *MsWHY5.1*, *MsWHY5.2,* and *MsWHY5.3* were located in mitochondria and chloroplasts, and *MsWHY3*, *MsWHY6*, *MsWHY7,* and *MsWHY8* were located in chloroplasts. These results indicate WHY proteins play different functions in different organelles.

**Table 1:** Basic information about members of the Whirly gene family in alfalfa.

Gene name	Gene ID	Length/aa	Molecular weight/D	PI	GRAVY	Subcellular localization
*MsWHY1*	MS.gene49569.t1	91	10,490.38	10.28	− 0.105	Mitochondria
*MsWHY2*	MS.gene032805.t1	290	32,491.09	5.82	− 0.32	Mitochondria
*MsWHY3*	MS.gene91941.t1	144	16,391.96	8.48	− 0.076	Chloroplast
*MsWHY4*	MS.gene025976.t1	190	20,866.89	9.46	− 0.085	Chloroplast; mitochondria
*MsWHY5.1*	MS.gene024508.t1	223	24,775.3	9.38	− 0.215	Chloroplast; mitochondria
*MsWHY5.2*	MS.gene050578.t1	223	24,775.3	9.38	− 0.215	Chloroplast; mitochondria
*MsWHY5.3*	MS.gene22868.t1	223	24,775.3	9.38	− 0.215	Chloroplast; mitochondria
*MsWHY6*	MS.gene25421.t1	194	21,709.85	9	− 0.267	Chloroplast
*MsWHY7*	MS.gene72950.t1	266	29,413.39	9.3	− 0.383	Chloroplast
*MsWHY8*	MS.gene63226.t1	268	29,724.73	9.3	− 0.388	Chloroplast

To better understand the molecular function of MsWHY proteins, we usedSPOMA online software to predict the secondary structures of 10 MsWHY proteins (Table 2). We found that random coil is the main component of the secondary structure of MsWHY proteins, which is more than 55% in most MsWHY proteins (except MsWHY2 and MsWHY3). However, the secondary structure α-helix of MsWHY2 and MsWHY3 proteins accounted for a large proportion (more than 35%), while the proportion of other proteins MsWHY proteins was small (MsWHY7 had the lowest 12.78%). The proportion of extended chains in MsWHY protein structure was less than 25% (except MsWHY4).

**Table 2:** Prediction of Whirly protein secondary structure in Alfalfa.

Gene name	Gene ID	Alpha helix (Hh)	Extended strand (Ee)	Random coil (Cc)
*MsWHY1*	MS.gene49569.t1	25(27.47%)	15(16.48%)	51(56.04%)
*MsWHY2*	MS.gene032805.t1	114(39.31%)	44(15.17%)	132(45.52%)
*MsWHY3*	MS.gene91941.t1	54(37.50%)	26(18.06%)	64(44.44%)
*MsWHY4*	MS.gene025976.t1	37(19.47%)	48(25.26%)	105(55.26%)
*MsWHY5.1*	MS.gene024508.t1	49(21.97%)	49(21.97%)	125(56.05%)
*MsWHY5.2*	MS.gene050578.t1	49(21.97%)	49(21.97%)	125(56.05%)
*MsWHY5.3*	MS.gene22868.t1	49(21.97%)	49(21.97%)	125(56.05%)
*MsWHY6*	MS.gene25421.t1	35(18.04%)	47(24.23%)	112(57.73%)
*MsWHY7*	MS.gene72950.t1	34(12.78%)	60(22.56%)	172(64.66%)
*MsWHY8*	MS.gene63226.t1	35(13.06%)	54(20.15%)	179(66.79%)

### Phylogenetic analysis of *MsWHYs*

**Figure 1:**
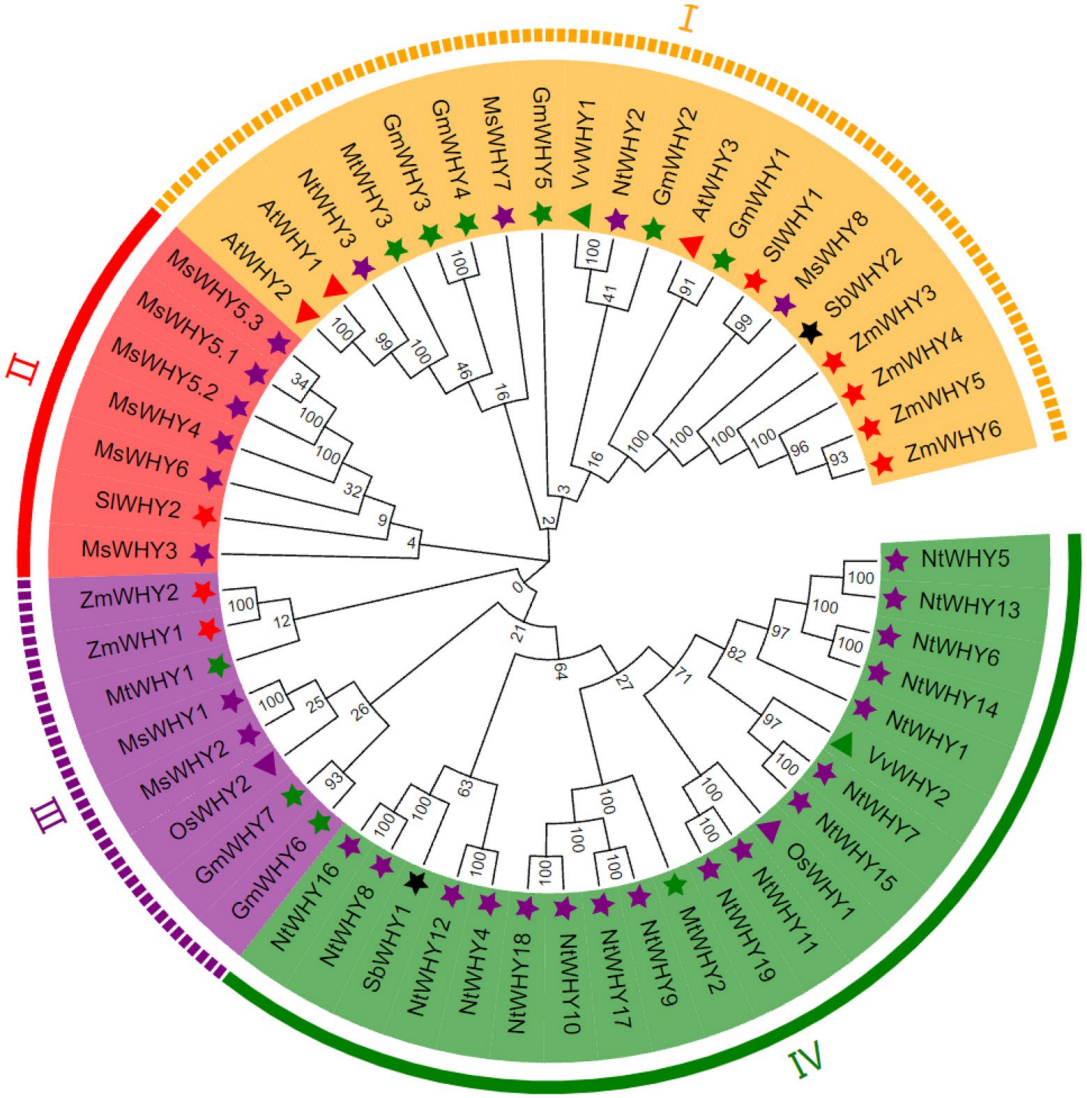
Phylogeny of the *MsWHY* gene family. The phylogenetic tree of the *MsWHY* gene family was constructed using all 46 *WHY* genes of *Arabidopsis thaliana* (At), *Oryza sativa* (Os), *Nicotiana tabacum* (Nt), *Medicago sativa* (Ms), *Zea mays* (Zm), *Medicago truncatula* (Mt), *Glycine max* (Gm), *Solanum Lycopersicum* (Sl), *Vitis vinifera* (Vv), and *Sorghum bicolor* (Sb) as outgroup. The subfamilies of the *MsWHY* gene family are indicated by I, II, III, and IV. The number nearby each cluster indicates the bootstrap confidence of the cluster in percentage (%).

The molecular evolution of the WHY family is decided mainly by the evolution of increasingly sophisticated organs in plants^52^. To investigate the phylogenetic relationships of *WHY* gene families in alfalfa, an unrooted phylogenetic tree was constructed from the alignment of full-length WHY proteins of maize, *Arabidopsis thaliana*, rice, sorghum, grape, tobacco, soybean, barrel medic, and tomato (data in S5 Table). The results showed that 46 *WHY* genes are grouped into four sub-families and correspondingly named categories I to IV based on a previous study (Fig. 1). The number of members in each subgroup is unevenly distributed. Among them, the subfamily II had the most members, and they were *MsWHY3*, *MsWHY4*, *MsWHY5.1*, *MsWHY5.2*, *MsWHY5.3*, and *MsWHY6*; Subfamily I and III each had two members, and the members were *MsWHY7*-*MsWHY8* and *MsWHY1*-*MsWHY2*. Alfalfa subfamily II family members were closely related to *SlWHY2*. Alfalfa subfamily I family members were closely related to *AtWHY1*-*AtWHY3*, *NtWHY2*-*NtWHY3*, *GmWHY1*-*GmWHY5*, *VvWHY1*, *MtWHY3*, *SbWHY2*, *SlWHY1*, and *ZmWHY3*-*ZmWHY6* and were properly grouped. Alfalfa subfamily III family members were associated with *ZmWHY1*-*ZmWHY2*, *MtWHY1*, *OsWHY2*, and *GmWHY6*-*GmWHY7*. For proteins that are closely related, it is speculated that they have similar or similar biological functions. At the same time, it was found that the *WHY* genes of monocotyledons clustered into small branches, indicating that the *WHY* gene families of monocotyledons and dicotyledons are in the evolutionary process.

### Analysis of the chromosome location, gene duplication, and synteny of the *MsWHYs*

**Figure 2:**
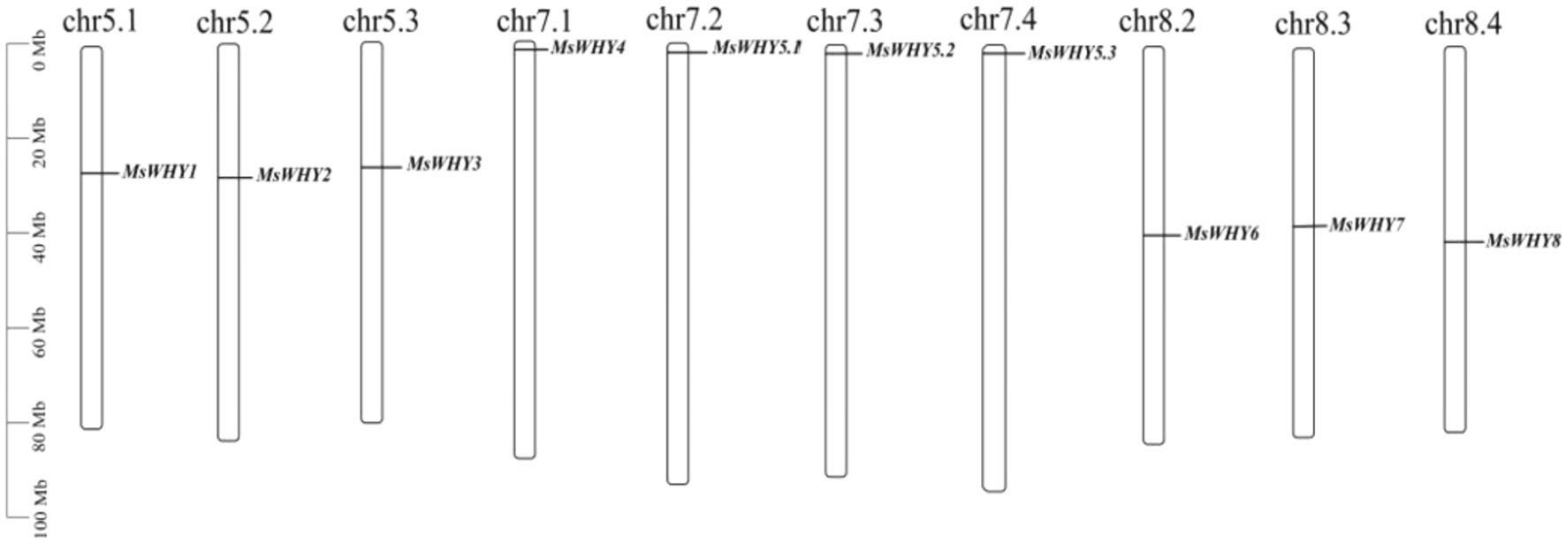
The distribution of *MsWHY* genes on 10 chromosomes in alfalfa. The scale (Mb) bar of the left displays the length of alfalfa chromosomes. The number of the chromosome is shown at the top of the chromosome.

According to the annotation information in the alfalfa genome, we found that 10 *MsWHY* genes are distributed on 10 chromosomes of alfalfa (Fig. 2). And the *MsWHY1*, *MsWHY2,* and *MsWHY3* genes were distributed on MsChr5.1, MsChr5.2, and MsChr5.3 chromosomes, respectively. *MsWHY4*, *MsWHY5.1*, *MsWHY5.2* and *MsWHY5.3* were all distributed at the endpoints of MsChr7.1, MsChr7.2, MsChr7.3 and MsChr7.4, respectively. *MsWHY6*, *MsWHY7* and *MsWHY8* genes were distributed on MsChr8.2, MsChr8.3 and MsChr8.4 chromosomes, respectively.

**Figure 3:**
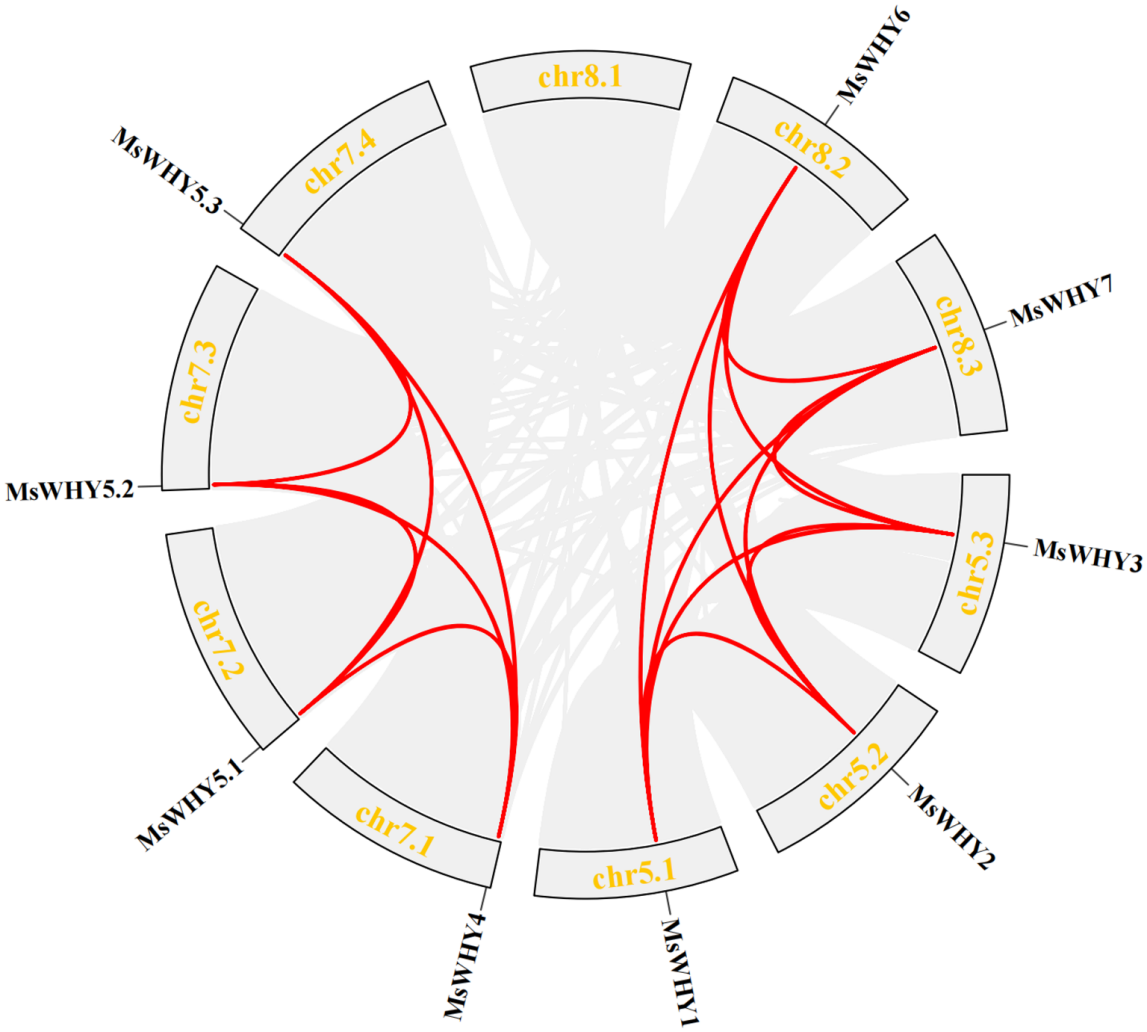
Syntenic relationship of *MsWHYs*. The number on the fragments represents the positions on the corresponding chromosomes. The *MsWHYs* involved in segmental duplications in the *MsWHY* gene family are mapped to their respective locations of the alfalfa genome in the circular diagram. The red lines represent the segmental duplication pairs between the *MsWHYs* and the gray lines represent the segmental duplication pairs in the whole alfalfa genome.

In the context that alfalfa is an autotetraploid with large genomes, we further examined the duplication events in the *MsWHY* gene family. Repeat events include segment repetition and tandem repetition. Segmental duplications are long DNA fragments that are nearly identical and present in distant chromosome locations^53^. They occur most commonly in plants because most plants are diploidized polyploids, and retain a large number of duplicated chromosomes in their genomes^54^. However, tandem duplication occurred mainly in chromosome recombination region^55^. To better understand the expansion patterns of *MsWHY* genes, we performed a colinear analysis of *MsWHY* genes and found that nine *MsWHY* genes were colinear with multiple genes in the family, such as *MsWHY1* and *MsWHY2*, *MsWHY3*, *MsWHY6*, and *MsWHY7* (Fig. 3). A total of 16 pairs of genes were found to have a co-linear relationship. All nine genes were copied in fragments, suggesting that fragment repetition plays a key role in the expansion of the gene family. To elucidate the selective pressure on the duplicated *MsWHY* genes, we calculated the non-synonymous (Ka) and synonymous substitutions (Ks), and the Ka/Ks ratios for the 8 *MsWHY* gene pairs (Table 3). The value of Ka/Ks = 1 denotes that genes experienced a neutral selection; < 1 suggests a purifying or negative selection; and > 1 indicates a positive selection^56^. The duplication events were calculated (T) using the formula T = Ks/2λ (λ represents the estimated clock-like rate of synonymous substitution, which is 1.65 × 10^−8^ substitutions/synonymous site/year for cereals)^57^. Our analysis revealed 8 segmental duplication pairs in *MsWHYs* (Table 3), with no tandem duplicate pairs. This is because alfalfa is homotetraploid and the 10 *MsWHYs* are located on different chromosomes. Furthermore, their Ka/Ks values vary from 0 to 0.8530, which are all less than 1, indicating that they are subject to purification selection during the evolution process. The dates of these segmental duplication pairs were 0.438 to 20.051 million years ago. Thus, these results indicate the conserved evolution of *MsWHY* genes.

**Table 3:** The duplication events of *MsWHYs* identified in alfalfa.

No	Sequence	Duplication type	Ka	Ks	Ka/Ks	Date (Millions of years ago)
1	*MsWHY2 & MsWHY1*	Segmental	0.1834	0.3008	0.6098	20.051
2	*MsWHY3 & MsWHY2*	Segmental	0.0518	0.1037	0.4996	6.91
3	*MsWHY5.1 & MsWHY4*	Segmental	0.0257	0.0302	0.8530	2.012
4	*MsWHY5.2 & MsWHY5.3*	Segmental	0	0.0066	0	0.438
5	*MsWHY5.2 & MsWHY5.1*	Segmental	0	0.0066	0	0.438
6	*MsWHY5.2 & MsWHY4*	Segmental	0.0257	0.0379	0.6789	2.527
7	*MsWHY5.3 & MsWHY4*	Segmental	0.0257	0.0302	0.8530	2.012
8	*MsWHY6 & MsWHY7*	Segmental	0.0414	0.0778	0.5325	5.186

### Gene structure and conserved motif analysis

**Figure 4:**
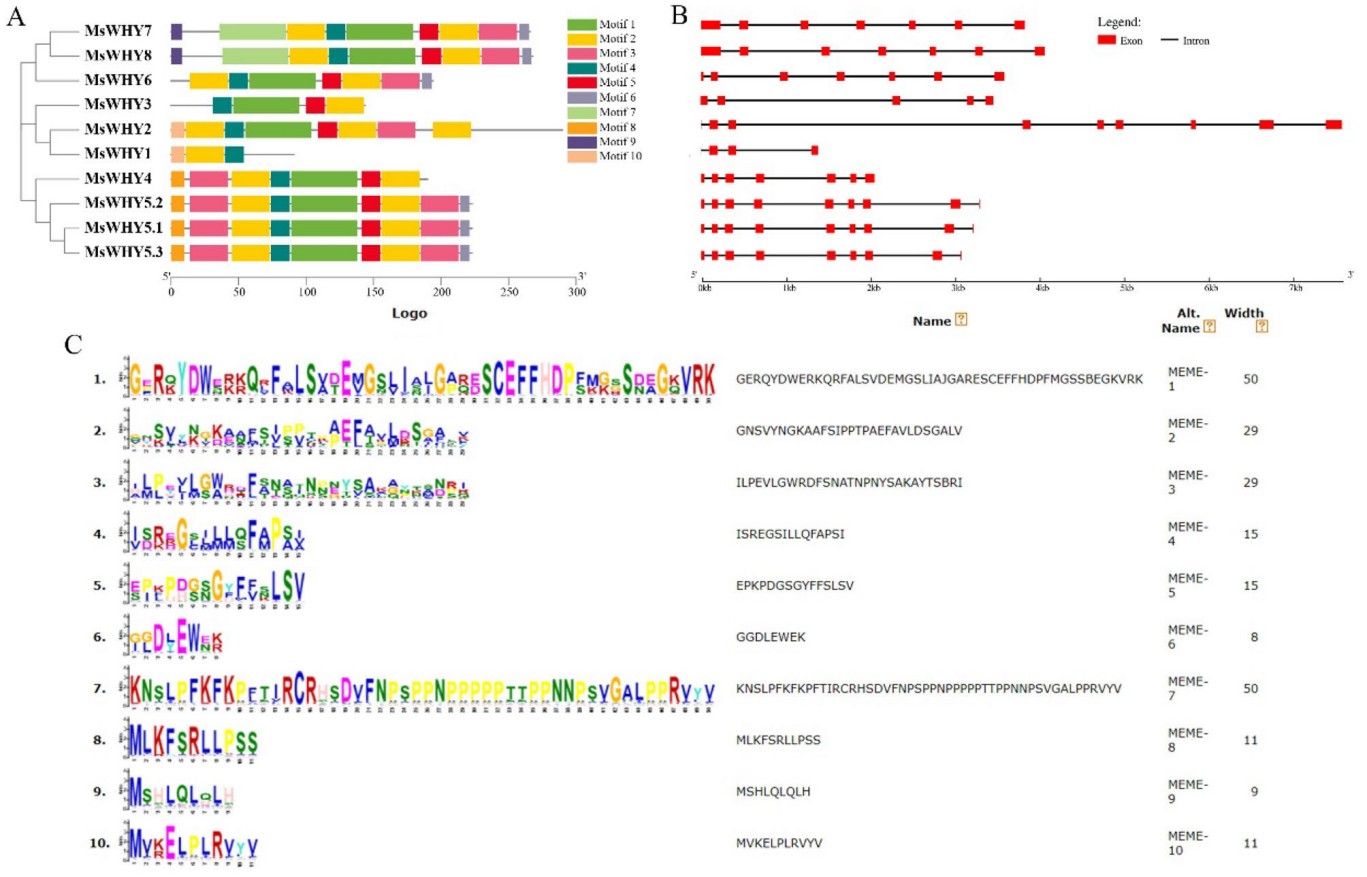
The gene structure and conserved motifs analysis of the *MsWHY* genes. (**A**): Colored boxes representing different conserved motifs having different sequences and sizes. (**B**): Exon–intron organization of the *MsWHY* genes. (**C**): Conserved motif. The overall height of each stack represents the degree of conservation at this position, while the height of the individual letters within each stack indicates the relative frequency of the corresponding amino acids.

To understand the structural characteristics of the *MsWHY* genes, the exon–intron structures, and conserved motifs of *MsWHY* genes were analyzed (Fig. 4). We observed that the structure of the exons and introns of the *MsWHY* genes of alfalfa were different among different subfamilies but relatively conserved within the same subfamily. Gene structure (Fig. 4B) analysis of *MsWHYs* showed that the number of introns varied from 3 to 8. Of these, *MsWHY4*, *MsWHY6*, *MsWHY7,* and *MsWHY8* all have 6 introns, while *MsWHY2*, *MsWHY5.1*, *MsWHY5.2*, and *MsWHY5.3* each contain 8 introns. *MsWHY1* and *MsWHY3* have fewer introns, 3 and 4, respectively. In addition, introns and exosomes in the *MsWHY1*, *MsWHY2*, and *MsWHY3* gene structures of the same branch were significantly different, especially in the number and length of introns in the *MsWHY2*. Although the introns of *MsWHY* genes were different, the members with the highest homology had similar gene structure, intron length, and the same number of exons, such as *MsWHY5.1 MsWHY5.2*, and *MsWHY5.3*. In addition, we also elucidated the conserved base sequence of the *MsWHY* genes using the MEME (Multiple Em for Motif Element) online servers. Finally, 10 conserved motifs were identified in *MsWHYs* (Fig. 4A,C). Motifs owned by or shared by most members of a gene family may be indispensable components of a gene family and have important functions or structures. We found although the conserved motifs of 10 *MsWHYs* are different in composition, all 10 *MsWHYs* contain motif 2 and motif 4, indicating that motif 2 and motif 4 play an extremely important role in the *MsWHYs MsWHY5.1*, *MsWHY5.2*, *MsWHY5.3*, *MsWHY7,* and *MsWHY8* contain 9 motifs, *MsWHY4* and *MsWHY6* contain 7 motifs, *MsWHY1*, *MsWHY2,* and *MsWHY3* contain 3, 8 and 4 motifs, respectively. Furthermore, the conserved motifs of *MsWHY5.1*, *MsWHY5.2*, and *MsWHY5.3* were identical, as were the conserved motifs of *MsWHY7* and *MsWHY8*, suggesting that they may have similar molecular functions. This prediction could lead to the discovery of new members of the *MsWHY* gene family.

### Promoter cis-acting elements analysis of *MsWHYs*

**Figure 5:**
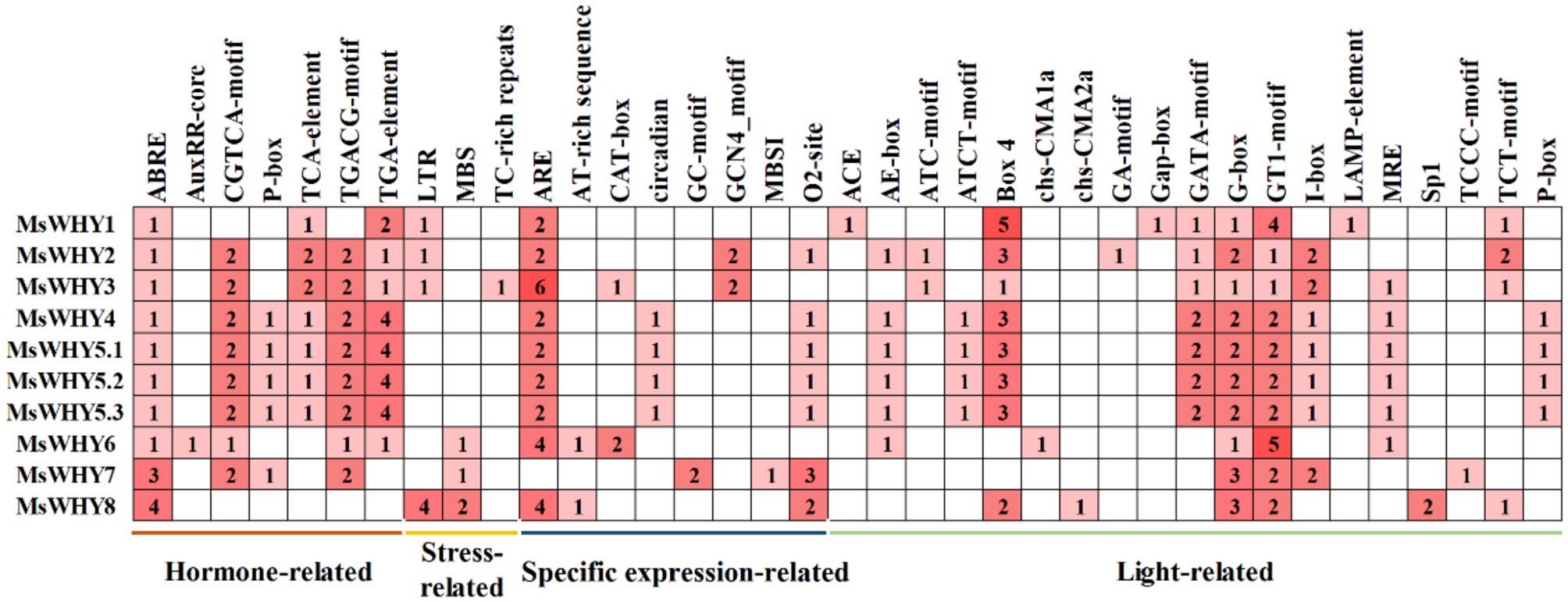
Putative cis-acting regulatory elements (CAREs) of the *MsWHY* gene family. The CAREs analysis was performed with the 2 kb upstream region using the PlantCARE online server. Hormone-responsive elements, stress-responsive elements, specific expression-related elements, and light-responsive elements are shown in different colors.

Promoter cis-acting elements are important transcription initiation binding regions of transcription initiation factors and play an important role in regulating gene expression. To further analyze the possible biological functions, we used the 2.0 kb sequence upstream of the MsWHY gene’s promoter to predict the regulatory elements of cis action through the Plant CARE website (Fig. 5). It is speculated that there are many cis-regulatory elements related to transcription, cell cycle, light, hormone, and stress response in the *WHY* genes promoter region of alfalfa, some of which are related to root specificity, leaf morphology specificity, seed specificity, and meristem specificity. In addition, we also found 7 elements related to hormone signaling pathways, ABRE, AuxRR-core, CGTCA-motif, P-box, TGACG-motif, TGA-element, and TCA-element. These cis-elements are involved in methyl jasmonate (MeJA), abscisic acid (ABA), salicylic acid (SA), gibberellin (GA), and auxin (IAA) metabolism regulation. In addition to *MsWHY1* and *MsWHY8*, the remaining 8 *MsWHYs* contained methyl jasmonate response elements (TGACG-motif and CGTCA-motif), and 10 *MsWHYs* all had ABRE response elements, which indicates that most *MsWHYs* can participate in JA and ABA-mediated signaling pathways. 3 cis-regulatory elements associated with response to external or environmental stresses were also present. This category includes a low-temperature responsive element (LTR), drought-inducibility element (MBS), and defense and stresses responsive element (TC-rich repeats). In the stress-related expression, the genes related to low temperature were *MsWHY1*, *MsWHY2*, *MsWHY3*, and *MsWHY8*. At the same time, *MsWHY6*, *MsWHY7*, and *MsWHY8* were related to drought. In addition, 8 *cis*-acting elements associated with tissue-specific expression were identified, anaerobic induction elements (ARE, GC-motif), AT-rich sequence, CAT-box, circadian control element (circadian), GCN4-motif, MBSI, and O2-site. It should be noted that all *MsWHYs* contain components related to light response, and all 10 *MsWHY* genes contain G-box and GT1-motif. The expression of these genes might be regulated by phytohormones, diverse light-responsiveness cis-elements, defense signaling transduction, and abiotic stresses during alfalfa growth.

### Protein interaction network diagram and three-dimensional structure prediction analysis

**Figure 6:**
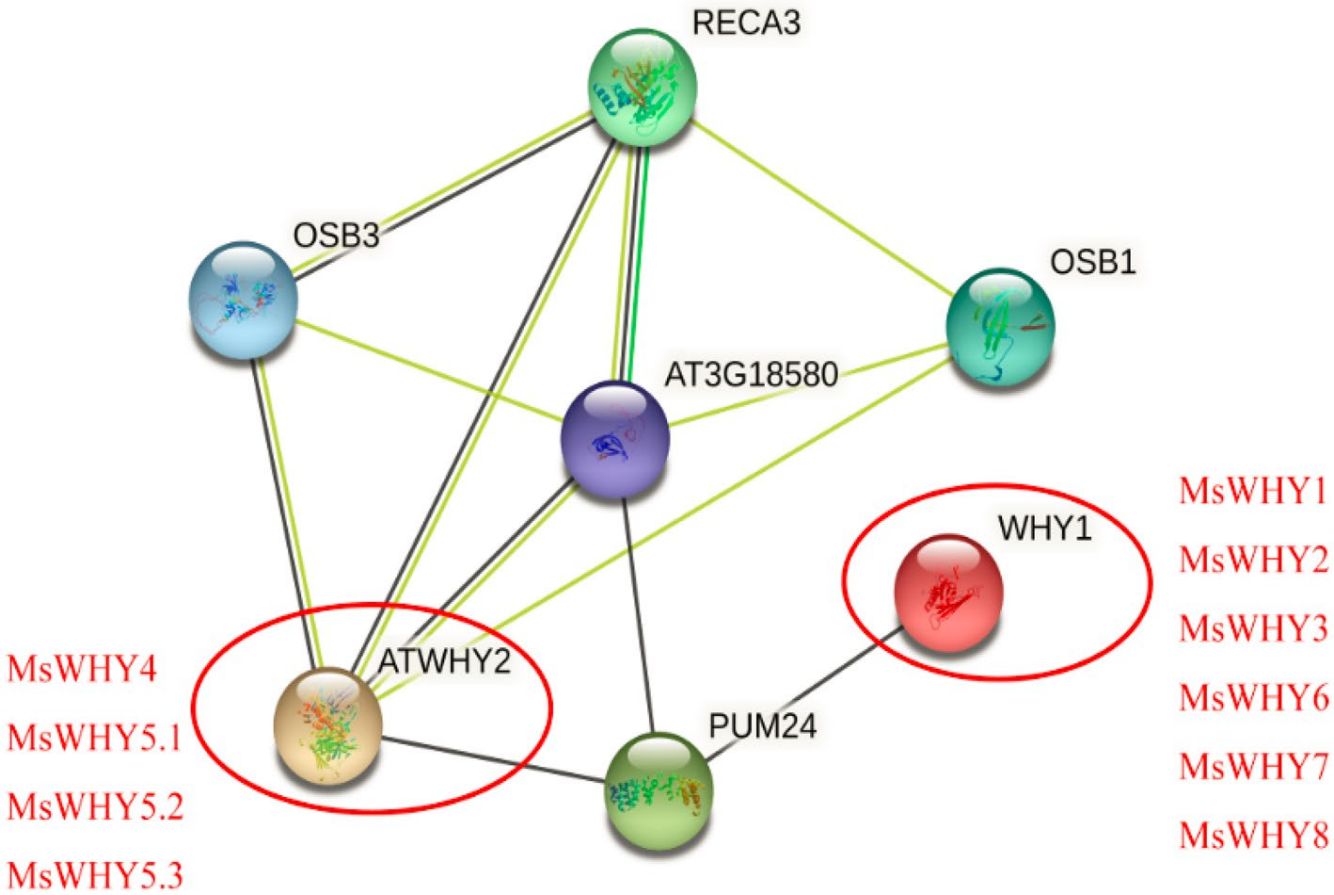
Protein–protein interaction analysis of MsWHYs proteins. Protein–protein interaction network produced by STRING, where each node represents a protein and each edge represent an interaction.

Using protein network interactions to connect unknown functional proteins to protein interaction networks will contribute to a further understanding of the rich biological functions of proteins and the dynamic regulatory networks among various biomolecules. Therefore, in this study, the model plant *Arabidopsis thaliana* was used as the background to predict the physical and chemical properties of the WHY protein and its potential function-related interacting proteins (Fig. 6).

**Figure 7:**
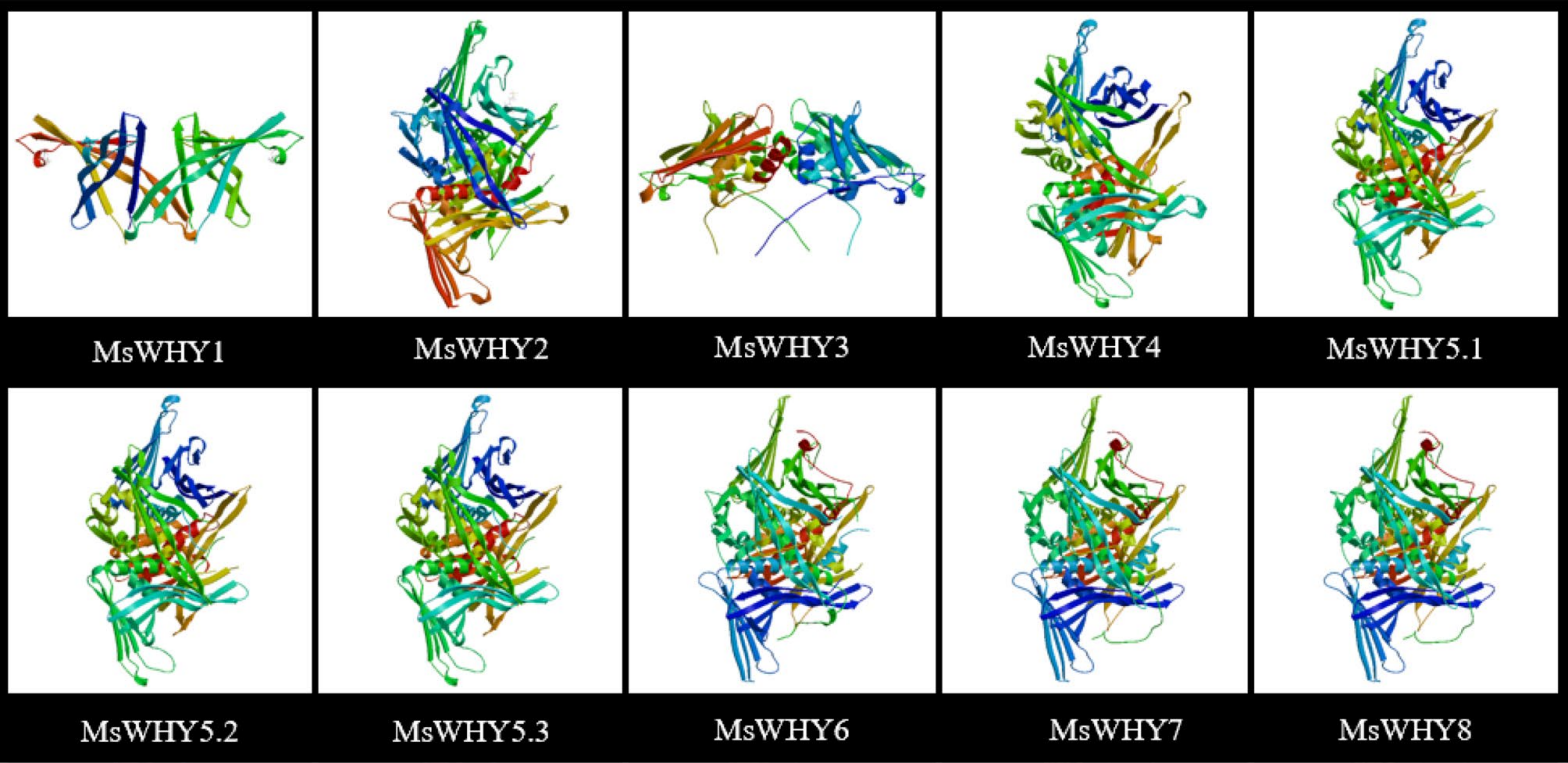
Three-dimensional structures of MsWHY proteins.

The expected edge number of our interaction network graph is 10, the average local clustering coefficient is 0.803, and the protein–protein interaction enrichment *P* value is < 0.00769, so we consider the results to be reasonable. We identified two WHY functional molecules and five potential interacting proteins directly related to MsWHY proteins (Fig. 6). They are OSB1, RECA3, OSB3, AT3G18580, and PUM24. ATWHY2 regulates leaf premature senescence, pollen tube activity, and pod development. WHY1 plays an important role in chloroplast and nucleus. In the nucleus, WHY1 is involved in the regulation of plant disease resistance, stress resistance, and senescence. Therefore, we can infer that the functions of 10 WHYs transcription factors in alfalfa are similar to those of the above two Arabidopsis transcription factors. MsWHY1, MsWHY2, MsWHY3, MsWHY6, MsWHY7 and MsWHY8 have similar functions to WHY1, and MsWHY4, MsWHY5.1, MsWHY5.2 and MsWHY5.3 have similar functions to ATWHY2. In this study, the amino acid sequences of 10 members of the *MsWHY* gene family were modeled by 3D structural homology. The software Swiss-Model was used for online analysis, and the tertiary amino acid sequences of 10 members of the *MsWHY* gene family were highly similar (Fig. 7). Such as MsWHY4, MsWHY5.1, MsWHY5.2, and MsWHY5.3 have highly similar tertiary structures. In addition, the three-tiered structure of MsWHY6, MsWHY7, and MsWHY8 are highly similar. The three-stage structure of MsWHY1, MsWHY2, and MsWHY3 differed significantly. However, the tertiary structures are not identical, which may be related to α-helix, β-folding, and irregular criability. These similarities or differences may account for their similar or different functions.

### qRT-PCR analysis

**Figure 8:**
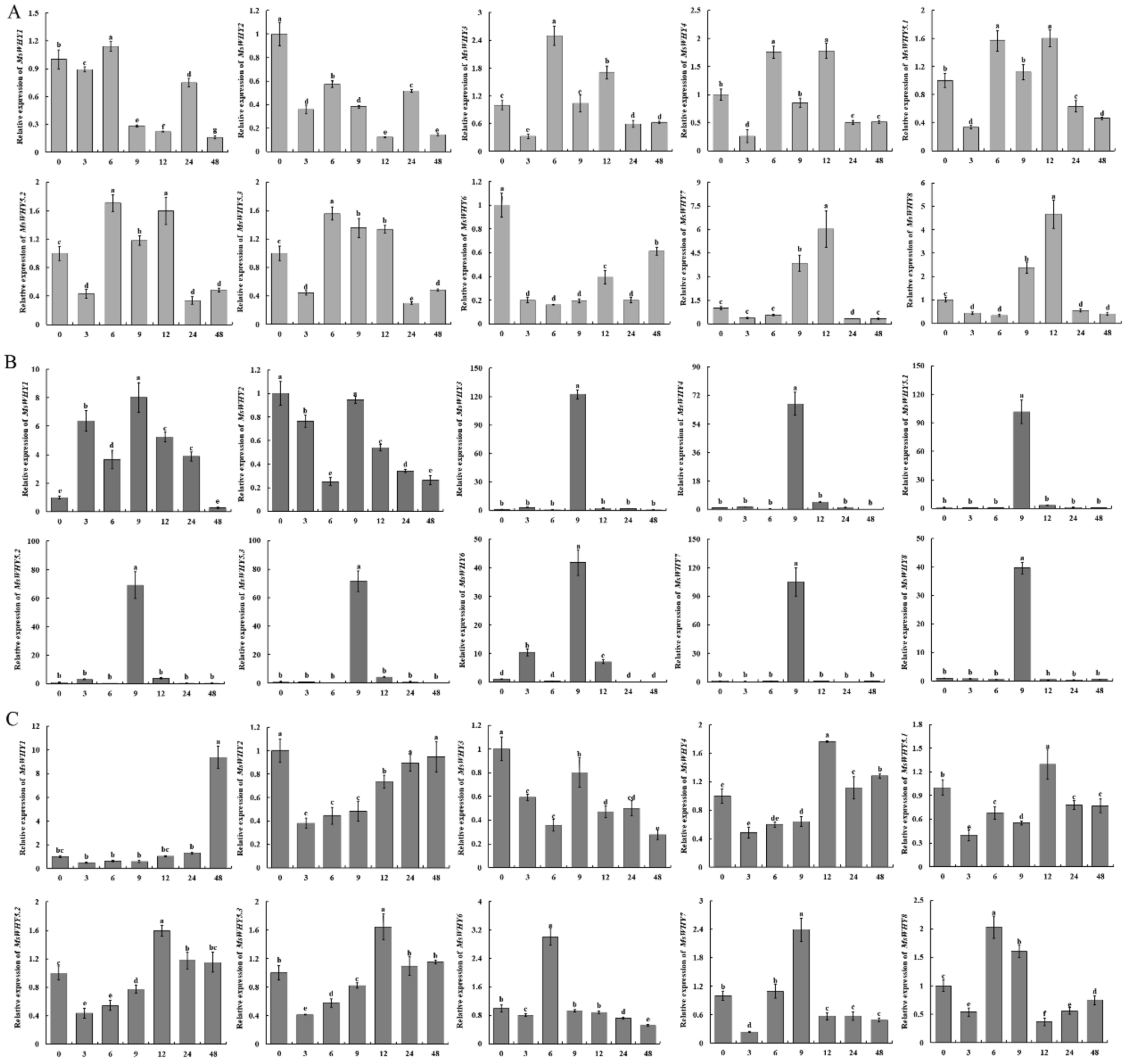
qRT-PCR expression analysis of *MsWHY* genes. Treatment time: 0 h, 3 h, 6 h, 9 h, 12 h, 24 h, and 48 h. (**A**): Expression analysis of *MsWHY* genes under PEG stress; (**B**): Expression analysis of *MsWHY* genes under NaCl stress. (**C**): Expression analysis of *MsWHY* genes under MeJA treatment. Error bars represent standard errors of three biological replicates. The different lowercase letters indicate significant differences at the *P* < 0.05 level.

To further determine the expression pattern of the *MsWHY* genes under abiotic stress (drought and salt) and hormone (MeJA) treatment, qRT-PCR was used to quantitatively detect the *MsWHY* gene’s expression under drought, salt, and MeJA treatment (S6 Table). Compared with the control (0 h), under drought stress (Fig. 8A), the expression levels of *MsWHY3*, *MsWHY4*, *MsWHY5.1*, *MsWHY5.2*, and *MsWHY5.3* were significantly up-regulated at three time points (6 h, 9 h, and 12 h). *MsWHY7* and *MsWHY8* were significantly up-regulated at two-time points (9 h and 12 h), indicating that the stress response of these genes was strong. However, the expression levels of *MsWHY1*, *MsWHY2,* and *MsWHY6* were lower under drought induction, indicating a weaker response to drought stress. Under salt stress (Fig. 8B), *MsWHY1*-*MsWHY8* all reached their highest expression levels at 9 h. Among them, the expression levels of *MsWHY3*-*MsWHY8* were similar, which gradually increased from 0 to 9 h and reached the highest point at 9 h. The expression levels gradually decreased during the subsequent experiment. However, the expression level of *MsWHY2* was lower than that of the control, indicating a weaker response to salt stress. After being sprayed with methyl jasmonate (Fig. 8C), *MsWHY1* and *MsWHY2* reached their highest expression levels at 48 h. *MsWHY3* and *MsWHY7* reached their highest expression levels at 9 h. *MsWHY4*-*MsWHY5.3* reached their highest expression levels at 12 h. *MsWHY6* and *MsWHY8* reached their highest expression levels at 6 h.

## Discussion

Members of the WHY protein family are found throughout the plant world, and WHY proteins play several important roles in plant development and stress tolerance. In particular, WHY1 regulates gene expression that encodes numerous housekeeping proteins and regulates plant development in response to biological and abiotic stress^1,5^. First, it acts as a transcription factor in the nucleus, regulating the expression of hormones such as ABA and SA, then as an organoid in organelle chloroplasts and mitochondria^5^. Barley WHY1 deficient plants showed delayed greening and delayed photosynthesis, suggesting WHY1 is necessary for chloroplast biogenesis^16,18^. In addition, deletion of WHY1 in maize leads to abnormal embryos and albinism^58^. This reported change in WHY-deficient plant phenotype indicates significant differences in WHY protein function across species. In this study, we found that WHY1 interacts with MsWHY1, MsWHY2, MsWHY3, MsWHY4, MsWHY6, MsWHY7, and MsWHY8 proteins, suggesting that the molecular functions of most *MsWHY* genes are similar to those of WHY1. Furthermore, the role of WHY1 as a transcription factor regulating leaf aging is well documented. RNAi-mediated loss of WHY1 function in barley, for example, affects aging and stress tolerance^26,27^. Manipulating WHY1 distribution between nucleus and chloroplast has been shown to alter senescence and cellular redox homeostasis^59^. Our study also found that ATWHY2 interacts with MsWHY4-MsWHY5.3 proteins, and we hypothesized that *MsWHYs* play an important role in regulating leaf aging, among others. Studies have shown that WHY2 triple locates in mitochondria, plasmids, and nuclei, and that overexpression of WHY2 in Arabidopsis leads to leaf aging and abnormal growth of longhorns, as well as changes in starch metabolism and expression of genes associated with aging^26^.

Phylogenetic tree results show that the closer the clustering relationship is, the more likely it is to have similar functions^60^. In the phylogenetic tree constructed in this study, WHY proteins could be divided into four subfamilies, among which I, II, and III subfamilies contained two, six, and two genes, respectively. By subcellular localization analysis of alfalfa WHY proteins, only *MsWHY1* and *MsWHY2* were located in mitochondria, *MsWHY3* and *MsWHY6*-*MsWHY8* were located in chloroplast, and the other four MsWHY proteins were located in mitochondria and chloroplast. The location of WHY proteins in different subcells may mean that their functions are also different^61^. Studies have shown that the WHY2 protein located in mitochondria is speculated to be involved in maintaining cell stability and regulating the transcription of the mitochondrial genome in mitochondria, thus playing an important role in plant growth and development, but more sufficient evidence is still lacking^62^. In chloroplasts, the first WHY1 protein identified is the chloroplast nucleoid binding protein pTAC1, which is involved in chloroplast genome protection and damage repair^63^. Gene classification, phylogeny, and subcellular localization analysis can help to study the function of similar gene families more accurately and conveniently^64^. The analysis of gene structure showed that the structure of the *MsWHY* gene in alfalfa was significantly different, with a maximum of 8 introns and the minimum of only 3 introns. The individual gene structure of the alfalfa WHY family was different, and the different exon–intron structures also contributed to the diversification of gene function^65^. MsWHY proteins motif in alfalfa is highly conserved. These conserved motifs determine the relatively conserved function of the *MsWHY* genes. These conserved motifs determine the relatively conserved function of the *MsWHY* genes. In particular, some genes are missing some motifs, which may be one of the reasons for the functional diversity of the *MsWHY* genes^66^.

Homologous genes distributed in farther locations are usually referred to as segmental duplication events, while those located together are considered as tandem duplication events^67^. Our analysis shows that gene replication plays a major role in gene family expansion. Moreover, both Ka/Ks ratios were less than 0, suggesting that replication of the *MsWHY* genes occurs through purification selection and that the corresponding MsWHY proteins are considered to be relatively conservative^68^. In addition, the predicted earliest dates of duplication events in the *MsWHY* genes segmental duplication pairs ranged from 0.438 to 20.051 million years ago, these results suggest that this is an ancient gene family. Promoter cis-acting element analysis indicated that *MsWHYs* may be involved in a variety of important biological processes, such as transcription, cell cycle, development, hormone, and biological/abiotic stresses (Fig. 5). The *MsWHY* genes of alfalfa are closely related to hormone pathway and stress response. The main hormones were MeJA, ABA, GA, SA, and IAA. Common stresses include drought and low temperature. Transcription factors play an important role in regulating plant growth and development^67^. Through the regulation of a transcription factor, it can achieve the regulation of multiple functional related genes, to achieve the purpose of improving plant traits^68^. In this study, bioinformatics methods were used to analyze the transcription factors of alfalfa *WHY* and provide favorable evidence for the verification of its related functions, which provides a sufficient basis for the cultivation of alfalfa in the future. Mitochondria chloroplast and nucleus are three organelles containing genetic information in plants. It is well known that maintaining the stable expression of genetic information DNA plays a crucial role in plant growth and development. WHY proteins are special proteins that can be located in both the nucleus and plastid^69^. WHY proteins are important in both the nucleus and plastid. Research shows that the WHY protein's structural characteristics and physiological and biochemical functions are very complicated. Analysis of the physicochemical properties of *MsWHYs* revealed that all 10 *MsWHYs* were hydrophilic proteins, and the subcellular localization of *MsWHYs* revealed that the *MsWHYs* gene is primarily located in mitochondria and chloroplasts, suggesting that it plays an important role in mitochondria and chloroplasts. The division of WHY proteins are critical to their function in plant growth, development, and defense. WHY proteins are synthesized in cytosols and transmitted to mitochondria and the chloroplast domain via their targeted signaling. Although many scholars have carried out comprehensive research on WHY protein, there are still many problems to be further studied^70^.

Gene expression patterns are significant clues for clarifying gene function. In this study, we analyzed expression profiles of the 10 *MsWHY* genes. The expression patterns of *MsWHYs* under different stress treatments were significantly different, indicating that the genes responded differently to different stresses. Many studies have reported an increase in WHY transcriptome levels in plants exposed to environmental stresses such as salt and drought stress^70,71^, heat^72^, oxidative stress, and infection with the fungus, *Botrytis cinera*^71,73^. Similarly, the application of exogenous hydrogen peroxide contributes to the accumulation of WHY1 protein in the chloroplast of Arabidopsis^59^. In contrast, citral, a naturally occurring phytotoxic aromatic compound in lemon fruits, reduced the expression of all *WHY* genes^74^. However, WHY2 transcripts, but not WHY1 transcripts, were increased in dehydrated chickpea seedlings^75^. WHY1 has been implicated in plant responses to biotic and abiotic stresses and has been shown to bind to the promoters of a variety of genes encoding proteins involved in stress tolerance, particularly those containing ERF elements^71^. In many reports, WHY1-dependent changes in gene expression or other WHY1 interactions in the nucleus have been associated with increased stress tolerance, such as enhanced levels of *SlWHY1* and *SlWHY2* transcripts during drought and salt stress^71^. In this paper, we conducted a series of analyses and research on the *WHY* genes of alfalfa and found that it can be induced expression under abiotic stress. Whether it has a certain protective effect on plants under abiotic stress and the specific mechanism of action still need to be solved gradually in the future.

## Conclusion

In this study, the phylogeny and diversification of *WHY* genes in alfalfa were investigated at different levels, including gene structures, evolutionary relationships, promoter cis-acting elements, and expression patterns. All 10 *MsWHY* genes were divided into 4 groups, and genes in the same group shared similar evolutionary features and expression patterns, implying potentially similar functions for *MsWHY* genes. *MsWHY* genes were distributed on 10 chromosomes of alfalfa, and the tertiary structure of amino acid sequence was similar, but not identical. There are cis-regulatory elements in the *MsWHY* genes promoter region related to hormones, stress, specific expression, and light. Collinearity analysis showed that a high proportion of the *MsWHY* genes might be derived from segmental duplications with purifying selection, providing insights into possible functional divergence among members of the *MsWHY* gene family. The physical and chemical properties of the MsWHY protein and the potential interaction proteins related to its function were predicted. qPCR analysis showed that *MsWHY* genes had different degrees of response to drought and salt stress and methyl jasmonate. The results obtained in this study will provide key ideas for further research on more functions of the *WHY* genes in alfalfa.

## Supplementary Information

Supplementary Information 1.

Supplementary Information 2.

Supplementary Information 3.

Supplementary Information 4.

Supplementary Information 5.

Supplementary Information 6.

## Data Availability

The genome-wide data of alfalfa is obtained from “Alfalfa Breeder’s Toolbox” (https://www.alfalfatoolbox.org/) and the Whirly protein sequences of Arabidopsis and other species data were downloaded from the Phytozome database( https://phytozome.jgi.doe.gov/pz/portal.html ).The original contributions presented in this study are included in the article/Supplementary Material, further inquiries can be directed to the corresponding authors.
