# Supplementary material for: Genome-wide identification, phylogenetic, and expression analysis under abiotic stress conditions of Whirly (WHY) gene family in Medicago sativa L
Source: Sci Rep. 2022 Nov 4;12:18676. doi: 10.1038/s41598-022-22658-3 (PMC9636397; doi:10.1038/s41598-022-22658-3)
Supplement: Supplementary file 1 — Supplementary Information 1. [file 41598_2022_22658_MOESM1_ESM.docx]

**Table 1** Fluorescence quantitative PCR primer

| Primer name | Forward primer（5’-3’） | Reverse primer（5’-3’） |
| --- | --- | --- |
| *MsWHY1* | CTCACGGTGTCTCCTGTACC | TTTGGCAGAACGAGGTGGAG |
| *MsWHY2* | TGCCATGCCTCTTAGGTTGG | CGAACAGTCCTGCTGTGGAT |
| *MsWHY3* | CTCACGGTGTCTCCTGTACC | CATATTGGCGAAAACCGGCA |
| *MsWHY4* | CTTGTTGTTGATCGTCGCGG | AATCTTGGGGACCAATGGCT |
| *MsWHY5.1* | CTTGTTGTTGATCGTCGCGG | AATCTTGGGGACCAATGGCT |
| *MsWHY5.2* | CTTGTTGTTGATCGTCGCGG | AATCTTGGGGACCAATGGCT |
| *MsWHY5.3* | CTTGTTGTTGATCGTCGCGG | AATCTTGGGGACCAATGGCT |
| *MsWHY6* | CTGCGAACGTGGATGTAAGC | GGATTAGCATTGTTCGCCGC |
| *MsWHY7* | AGCTTTGCCACCTAGGGTTT | AGCAGCAAACAACCATCCCT |
| *MsWHY8* | AGCTTTGCCACCTAGGGTTT | AGCAGCAAACAACCATCCCT |
| *GAPDH* | GGCTGCCATCAAGGAGGAAT | TCCAAGCTCAGCCTCATCAAG |
